# A60 ANXIETY AND DEPRESSION ARE ASSOCIATED WITH POOR ADHERENCE TO THE GLUTEN-FREE DIET, GREATER SYMPTOMS SEVERITY, AND REDUCED QUALITY OF LIFE IN TREATED CELIAC DISEASE PATIENTS

**DOI:** 10.1093/jcag/gwae059.060

**Published:** 2025-02-10

**Authors:** F J Echagüe, A K Verma, R Leong, A Aman, J Blom, V Noejovich, R Daca, D Armstrong, M Pinto-Sanchez

**Affiliations:** McMaster University, Hamilton, ON, Canada; Medicine, McMaster University, Hamilton, ON, Canada; Medicine, McMaster University, Hamilton, ON, Canada; McMaster University, Hamilton, ON, Canada; McMaster University, Hamilton, ON, Canada; Medicine, McMaster University, Hamilton, ON, Canada; McMaster University, Hamilton, ON, Canada; McMaster University, Hamilton, ON, Canada; McMaster University, Hamilton, ON, Canada

## Abstract

**Background:**

Studies have reported an association between celiac disease (CeD) and anxiety and depression. However, how this impacts symptoms and disease management is unknown.

**Aims:**

To assess 1) the prevalence of anxiety and depression in adult patients with celiac disease and 2) the association of anxiety and depression with CeD outcomes including symptoms, disease activity, treatment adherence and quality of life.

**Methods:**

Consecutive patients attending the adult Celiac Disease Clinic at a tertiary care hospital were enrolled in the Celiac Registry. Participants completed the gluten-free diet (GFD) adherence (CDAT), Quality of Life (CD-QoL), Celiac Symptom (CSI), and Anxiety and Depression Scale (HADS) questionnaires. Anxiety or depression were defined based on a HADS score ≥ 7 for each subscale. CDAT <13 was considered good adherence to the GFD. CSI score ≤30 was considered a low CeD-symptom burden. Continuous variables were expressed as median (IQR) and categorical variables as percentages.

**Results:**

From October 2020 to October 2024, 673 CeD patients, [38 (31) years] were enrolled in the study. Of these, 251 (37%) met the criteria for anxiety and 109 (16%) for depression. Anxiety and depression were associated with lower adherence to a gluten-free diet (anxiety OR=0.7, p<0.001; depression OR=0.5, p<0.001), reduced QoL (anxiety OR=1.07, p<0.001; depression OR=1.03, p<0.001) and increased celiac-related symptoms (anxiety OR=0.56, p<0.001; depression OR=0.2, p<0.001). A non-significant trend of increasing weight was observed in CeD patients with anxiety and depression. Regression analysis showed QoL, diet adherence, and weight changes were predictors for an increased risk of anxiety and depression in CeD patients.

**Conclusions:**

Anxiety and depression are common in CeD patients, and they are associated with poor adherence to a GFD, symptom control, and worse quality of life. Addressing mental health in CeD is crucial for the management of celiac disease and has the potential to improve celiac outcomes.

**Funding Agencies:**

